# Synthesis of Gold Nanoparticles Decorated with Multiwalled Carbon Nanotubes (Au-MWCNTs) via Cysteaminium Chloride Functionalization

**DOI:** 10.1038/s41598-019-42055-7

**Published:** 2019-04-05

**Authors:** Vu Duc Chinh, Giorgio Speranza, Claudio Migliaresi, Nguyen Van Chuc, Vu Minh Tan, Nguyen-Tri Phuong

**Affiliations:** 1 Department of Industrial Engineering, University of Trento, via Sommarive 9, 38123 Trento, Italy; 2 Institute of Materials Science, Vietnam Academy of Science and Technology, 18 Hoang Quoc Viet road, Cau Giay district, Hanoi, Vietnam; 3 Graduate University of Science and Technology, Vietnam Academy of Science and Technology, 18 Hoang Quoc Viet road, Cau Giay district, Hanoi, Vietnam; 4 Centre for Materials and Microsystems, Fondazione Bruno Kessler, via Sommarive 18, I-38123 Povo, Trento Italy; 5 Hanoi University of Industry, Bac Tu Liem, Hanoi, Vietnam; 6 Institute of Research and Development, Duy Tan University, Da Nang, 550000 Vietnam; 7 Department of Chemistry, Université de Montréal, Quebec, Canada

## Abstract

Gold nanoparticles (AuNPs) decorated CNTs are promising materials for photocatalytics and biosensors. However, the synthesis of AuNPs chemically linked to the walls of MWCNTs is challenging and toxic products such as thionylchloride (SOCl_2_) or [1-ethyl-3(dimethyl-amino) propyl] carbodiimide hydrochloride (EDAC) need to be used. This work reports a new approach to prepare gold nanoparticles decorated multiwalled carbon nanotubes (MWCNTs) by using cysteaminium chloride via the formation of a *Zwitterionic* acide-base bond. The grafting process consists of 3 mains steps: oxidation, thiolation and decoration of AuNPs on the surface of MWCNTs. The completion of each step has been verified out by both spectroscopic (Raman, UV-Vis, FT-IR) and Scanning Electron Miscroscopy (SEM). The chemical bonding states of synthesized products have been proven by X-ray photoelectron spectroscopy (XPS).

## Introduction

Since their discovery by Iijima and Ichihashi^1^, carbon nanotubes (CNTs) have attracted great interest from the scientific community, thanks to their unique structural, mechanical, and electronic properties^2–7^. Single wall carbon nanotubes (SWCNTs) can be described as a graphitic monolayer rolled up into a nanoscale-tube. Additional layers may be folded together to generate multiwalled carbon nanotubes (MWCNTs). The combination of Au-NPs with CNTs to create nanohybrid materials (AuNPs-CNT)^8–10^ has received considerable attention for numerous applications such as biosensors (DNA, proteins, glucose)^11^, gas sensors (oxygen, water vapor)^12^, toxicant sensors (arsenic III)^13^ and drug delivery^14,15^. The combination of two materials can generate new properties which are not present in their counterparts as described in our recent publications^16–19^.

Metal decorated CNTs can be obtained by various approaches such as physical deposition^20^ or wet chemical deposition^21,22^. The Au nanoparticles can be directly attached to CTNs by physical absorption without any chemical link between the two components, or they can be chemically linked together to form a stable structure^23,24^. The latter is commonly formed thanks to covalent bonds between modified CNTs surface and gold nanoparticles dealing with Π-Π stacking, hydrophobic and electrostatistic interactions. Because the surfaces of CNTs are inert, a chemical treatment is needed to make it more active to react with other chemical compounds. Regarding AuNPs chemically linked CNTs nanocomposites, it has been reported that there are various chemical methods to attach gold nanoparticles (NPs) onto MWCNTs^14,15,25–30^. Zhang *et al*.^16^ reported the preparation of Au-CTNs nanocomposites by growing Au nanoparticles on the surface of MWCNTs through UV irradiation. Other functionalization techniques have been successfully used for this purpose, e.g., gold/iron-oxide magnetic NPs-decorated CNTs (Au/MNP-CNTs) were successfully synthesized through a two-step method^20^. Oxidized CNTs coated with poly-(diallyl dimethylammonium) chloride have also been used as a template for gold NPs self-assembly^21,22^.

Many applications require covalent bonds to meet specific purposes. In the case of biosensors of biomolecules, a stable chemical link between AuNPs and CNTs is needed. This type of modification commonly proceeds via three main steps: chemical oxidation, activation and amidation reactions of CNTs. Kardimi *et al*.^31^ prepared the MWCNT hybrid nanomaterials by treating CNTs with a 3:1 H_2_SO_4_/HNO_3_ mixture, followed by an activation step with SOCl_2_ and a mixture of acyl chloride and ethylene diamine. The as-prepared MWCNTs were again modified with mercaptoacetic acid coated QDs^31^. Jiang *et al*.^32^ immobilized, bovine serum albumin (BSA) protein via a two-step process of diimide-activated amidation on MWCNTs. In the first step, carboxylated MWCNTs were activated by N-ethyl-N′-(3-dimethylaminopropyl) carbodiimide hydrochloride (EDAC) to create a stable active ester in the presence of N-hydroxysuccinimide (NHS). In the second step, the activated ester groups were reacted with the amine groups of the BSA to form an amide bond between the MWNTs and the protein. This two-step process avoids the intermolecular conjugation of proteins and guarantees the uniform attachment of proteins on carbon nanotubes.

This work aims to synthesis gold nanoparticles decorated with MWCNTs in which the grafting of hydrosilane (HS) on the oxidized MWCNTs will be carried out by a new approach, using MWCNTs-COO^−^/^+^H_3_N-CH_2_-CH_2_-SH *zwitterionic* reaction. This *zwitterionic* reaction has been done by coupling the negative MWCNTs-COOH^–^ with the positive, primary amine groups of cysteamine via an electrostatic interaction, followed by the decoration of AuNPs on the MWCNTs surface. This approach is simple and cost-effective for the CNTs functionalization and avoids the use of dangerous and toxic chemical agents such as thionylchloride (SOCl_2_)^33^ or [1-ethyl-3(dimethyl-amino) propyl] carbodiimide hydrochloride (EDAC)^34,35^. Furthermore, such ionic features may allow electrostatic interactions between MWCNTs and biological molecules and can serve as the basis for developing MWCNTs biological probes.

## Experimental

### Materials

The MWCNTs used in this study were synthesized from our institute (Laboratory of Carbon nano, Institute of Materials Science, Hanoi, Vietnam) as described in a previous work^36^. Cysteaminium chloride (Chemical Formula: HSCH_2_CH_2_NH_3_^+^/Cl^−^, molar mass: 113.61 g/mol, assay: ≥97.0%) and Trisodium citrate were purchased from Merck, Darmstadt, Germany. Chloroauric acid, purity 99.999% was from Sigma-Aldrich, Oakville, Ontario, Canada. Sulfuric acid and nitric acid were supplied by Prolabo chemical, VWR International, New York, USA.

### Synthesis methods

**Figure 1:**
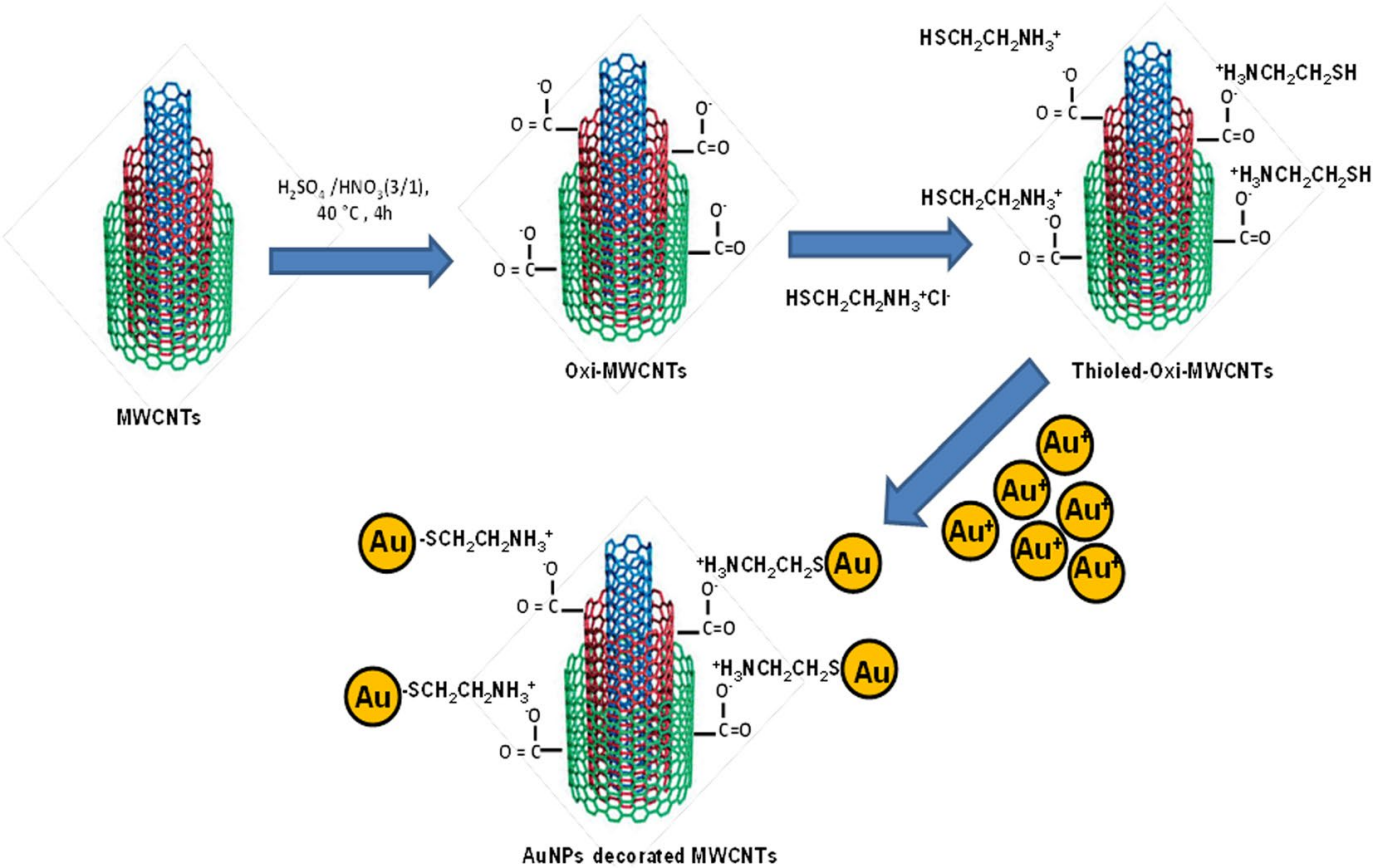
Schematic illustration for the preparation of Au-MWCNTs.

The decoration of gold nanoparticles on MWCNTs surface was carried out by three following steps: (1) Functionalization of carbon nanotubes surface; (2) grafting cysteaminium chloride by a thiolation reaction, and (3) decoration of gold nanoparticles by chemical covalent bonds. The synthesis procedure is schematically shown in Fig. 1.

#### Chemical oxidation of MWCNTs

0.2 g of MWCNTs were treated at 40 °C with 30 ml of a mixture of H_2_SO_4_ (98%)/HNO_3_ (62%) (3/1), in a flask connected to a condenser for 4 h, then left overnight in a 9:1 diluted water solution and finally washed and dried in an oven at 50 °C to obtain oxidized MWCNTs (Oxi-MWCNTs).

#### Thiolation of MWCNTs

22 mg of Oxi-MWCNTs, obtained in the step 1, were treated with a solution containing 27 mg of cysteaminum chloride in 10 ml distilled water at 90 °C for 30 h. After cooling to room temperature, the powder was washed with distilled water to remove excess of amine, followed by a centrifugation at 12000 rpm/min and then dried under vacuum for 4 h to obtain Thiol-Oxi-MWCNTs.

#### Preparation of gold NPs decorated MWCNTs

**Figure 2:**
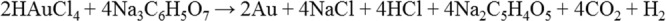
Reduction of Au (III) complex by sodium citrate.

The synthesis of Au-MWCNT nanocomposites was based on the reduction of Au (III) complex by sodium citrate, in which HAuCl_4_ solution (0.01 M) was added to the CNT aqueous suspension. Gold NPs were gradually formed as the citrate reduced Au^3+^ to Au^0^ according to following the reaction (Fig. 2).

The resulting suspension was sonicated for 30 min to promote the interaction of gold ions with the –SH on CNT surface. The solution was heated until 90 °C and then sodium citrate solution (2%) was added. The reaction was kept at these conditions for 1 h. The resultant nanocomposites were washed with distilled water and then separated by high speed centrifugation (12000 rpm). The final nanocomposite was dried at 80 °C overnight.

### Characterization

Morphology observations of pristine MWCNTs and Au-MWCNTs were carried out with a Field Emission Scanning Electron Microscope (FE-SEM), model S4800-Hitachi. XPS spectra were recorded by using an Axix DLD Ultra (Kratos - Manchester) with a monochromatized Al K_α_ radiation. Wide spectra were acquired at 160 eV pass energy which was reduced to 20 eV for the acquisition of the core lines, to increase the energy resolution. XPS peak fitting was performed using homemade software based on the R platform. The oxi-MWCNTs were analyzed by Micro Raman spectroscopy (XploRA; Horiba) at 532 nm (90 mW) or 785 nm (25 mW) excitation lines from a diode-pumped, solid-state laser to analyze the Raman vibrational bonds. The 100 mW laser was focused on the sample utilizing a 100x microscope objective leading to a laser spot size of ~1 μm. A charge coupled device (CCD) was used as a detector. The Raman spectra were acquired from 1000 to 2000 cm^−1^ with a spectral resolution of 2 cm^−1^. Fourier transform infrared (FTIR) spectra were collected with a Shimadzu IR Affinity-1S spectroscopy in the range of 400–4000 cm^−1^, with a spectral resolution of 4 cm^−1^ and 20 scans. The particle size distribution was measured using a Zetasizer instrument (6.20 version).

## Results and Discussion

### Chemical oxidation of carbon nanotubes

**Figure 3:**
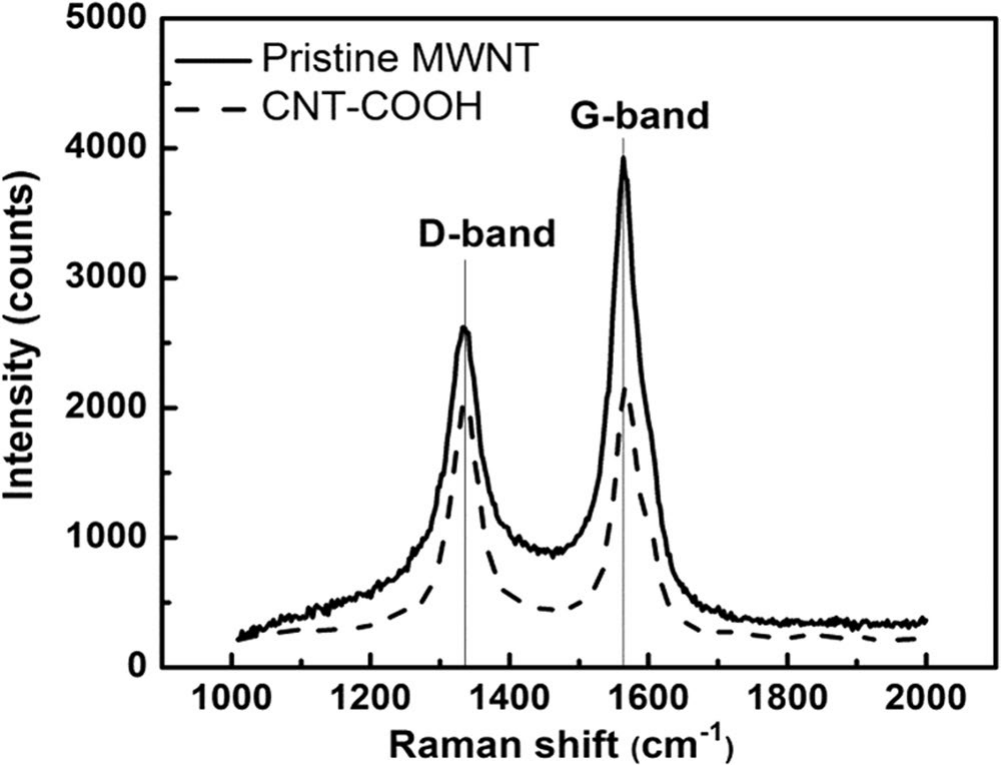
Raman spectra of pristine and oxidized MWCNTs.

The effect of chemical oxidation on the structural integrity of MWCNTs through a mixture of nitric and sulfuric acids has been firstly investigated by spectroscopic methods. This chemical treatment might lead to shortening and additional defect, generated in the graphitic network of MWCNTs. The Raman spectra of oxidized MWCNTs show two major peaks, located at 1334 and at 1564 cm^−1^ which were assigned to the D-band and to the G-band of CNTs, respectively (Fig. 3). The D-band at around 1334 cm^−1^ was associated to the presence of disordered graphitic domains and structural imperfections or impurities^37^.

The G-band at 1564 cm^−1^ band represents the E_2g_ stretching vibration of carbon atoms in the hexagonal crystalline structure^37^. The intensity ratio I_D_/I_G_, is sensitive to determine the degree of defects introduced by CNT chemical processing. Figure 2 shows a higher (I_D_/I_G_) ratio for the functionalized MWCNTs (0.94) compared to that of pristine MWCNTs (0.67) due to the incorporation of functional groups on the surface and thus leads to structural deformations. These results are in line with those reported in the literature^38^. The I_D_/I_G_ ratio of 1.62 after oxidation of MWCNT with HNO_3_/H_2_SO_4_ was reported by Tsukahara *et al*.^3^ and Dasyuk *et al*.^39^ have shown I_D_/I_G_ ratios of 1.45 and 1.01 after oxidation of MWCNTs with nitric acid under microwave assisted treatment and reflux in nitric acid for 48 h, respectively.

**Figure 4:**
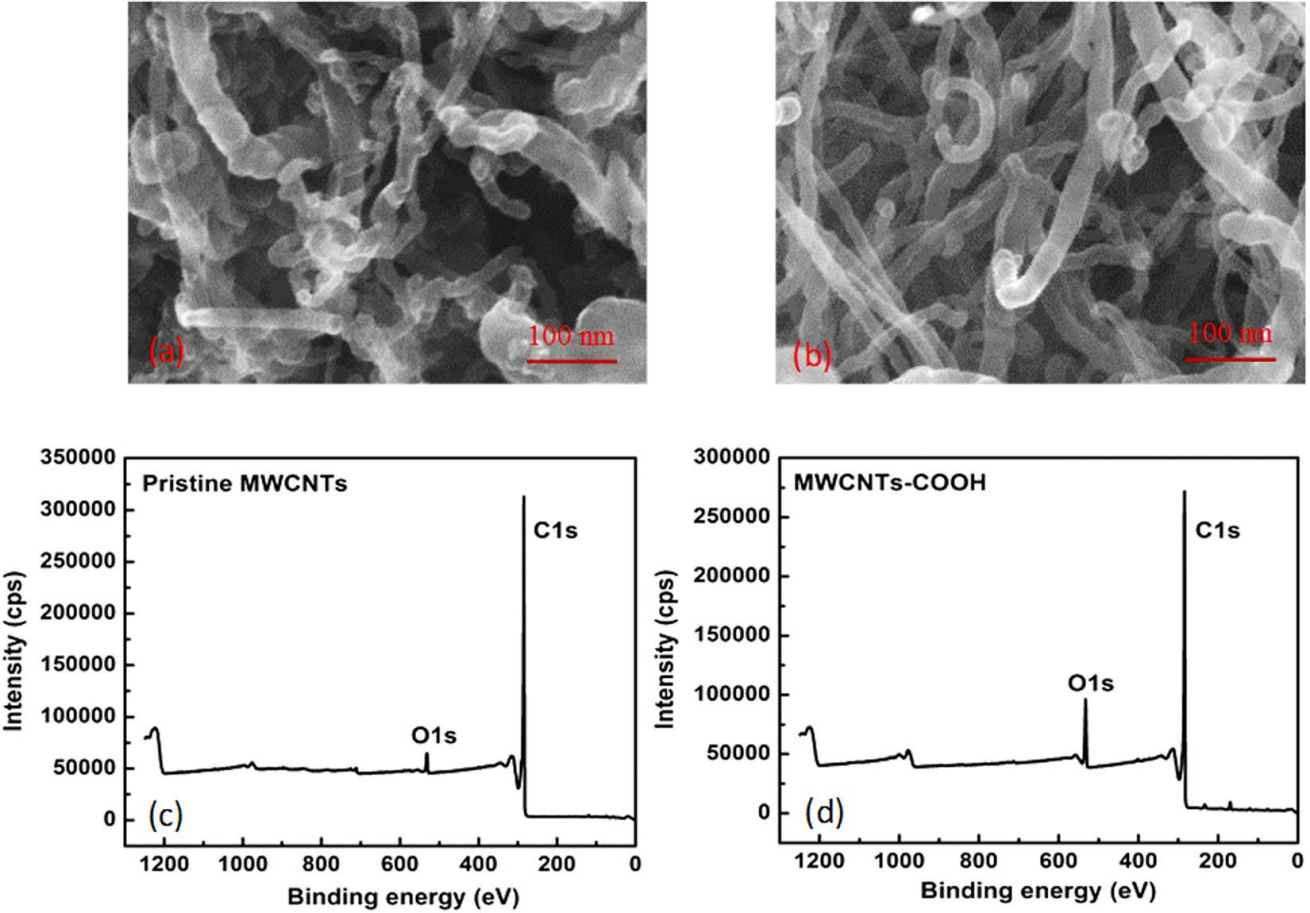
SEM images of (**a**) pristine MWCNTs; (**b**) Oxi-MWCNTs and the XPS survey spectra of (**c**) pristine MWCNTs and (**d**) Oxi-MWCNTs.

Figure 4a,b show SEM images of untreated and oxidized MWCNTs. Prior to oxidation, the surface of pristine MWCNTS shows the presence of some impurities on the surface that could result from the residual metallic catalysts during production process. The acidic treatment has successfully removed these impurities (Fig. 4b). Minati *et al*.^40^ also used mixture of H_2_SO_4_/HNO_3_ to cut CNTs into shorter open-ended pipes with the presence of large amounts of carboxylic and oxygen-containing groups at the open-end^40^. Zhang *et al*.^41^ argued that the treatment of CNTs by concentrated acidic solution is useful to prepare intercalated and exfoliated graphites with higher number of oxidation site on carbon atom. The oxidized sites might be formed on the side wall and at the end of tube^41^ and the defects appeared in the hexagonal crystalline structure allow the attack of strong acid mixtures to form oxygen- containing functional groups.

Figure 4c,d demonstrate the XPS survey spectra of the pristine MWCNTs and Oxi- MWCNTs. As-expected, atomic carbon is the main component of the MWCNTs. However, oxygen traces (about 2.6%) have been also detected in the pristine MWCNTs. This can be assigned to the initial contaminants in the pristine nanotubes during the production and purification steps. After the acidic treatment, the total amount of atomic oxygen has raised to 8.4%, due to the oxidation of the MWCNTs, while the carbon content is almost unchanged.

**Figure 5:**
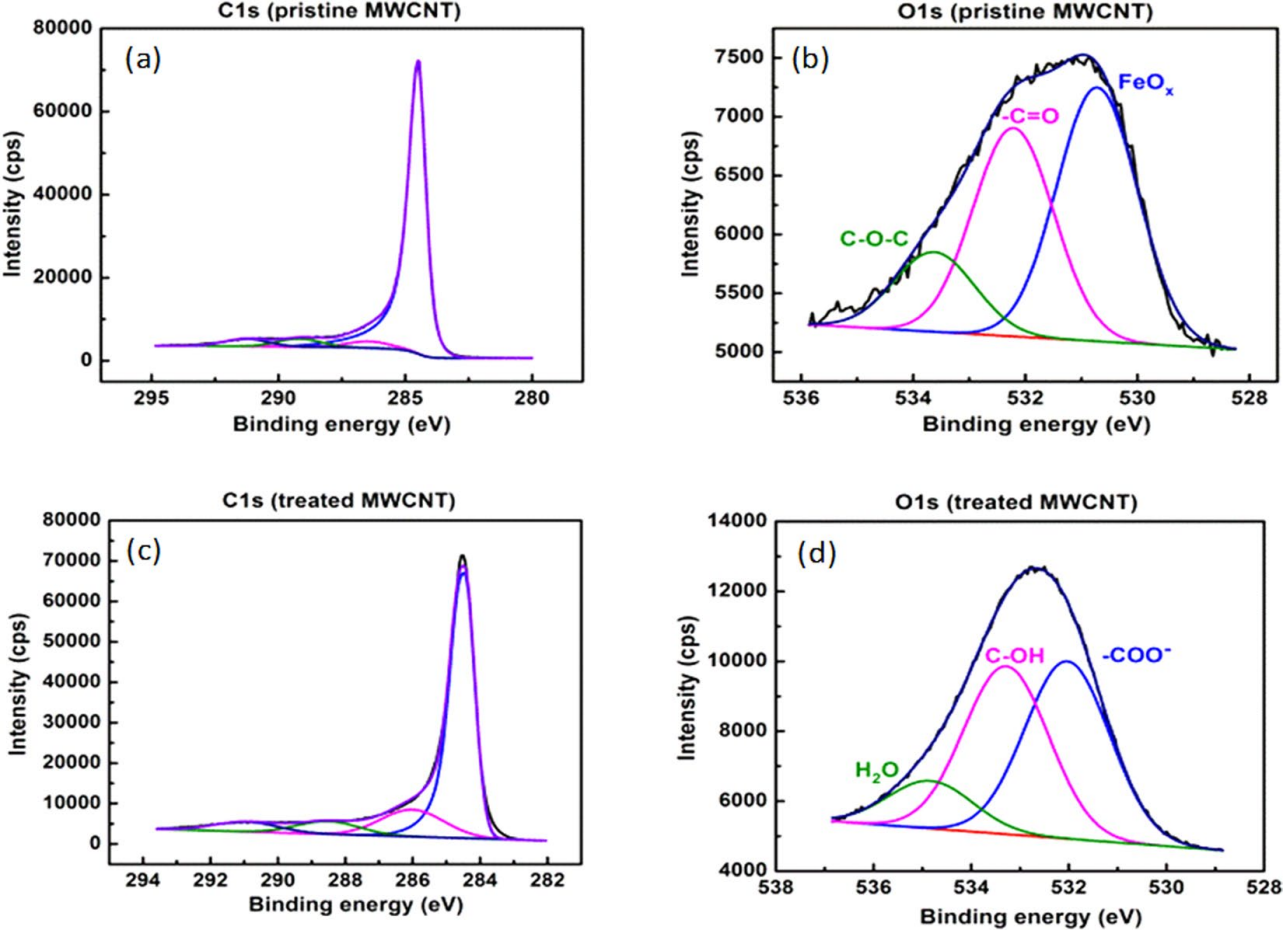
Deconvolution of: (**a**) C1s; (**b**) O1s of the pristine MWCNTs and (**c**) C1s, (**d**) O1s Oxi-MWCNTs.

The chemical process produces nanotubes with carboxylic terminated functional groups. A detailed analysis of covalent bonds formed during the acid attack is performed by acquisition of high resolution XPS spectra. The C1s core line of the pristine CNT shows the main peak position at 248.4 eV (Fig. 5a). The acid treated CNT (CNT-COOH) shows the same feature at 284.4 eV but also a new component at around 286 eV (assigned to C-OH bond) and a peak at 288.5 eV, assigned to the carboxylic groups (Fig. 5c). In the O1s core line analysis (Fig. 5b,d), the more intense peak located at 532 eV can be assigned to oxygen species in carboxylic group (C=O), while other components refer to the presence of C-OH bonds of carboxylic group and residual H_2_O, respectively. These peaks become more important after the implantation of oxygen containing groups by acidic treatment.

### Functionalization of the carbon nanotubes with thiol groups by acid-base reactions

**Figure 6:**
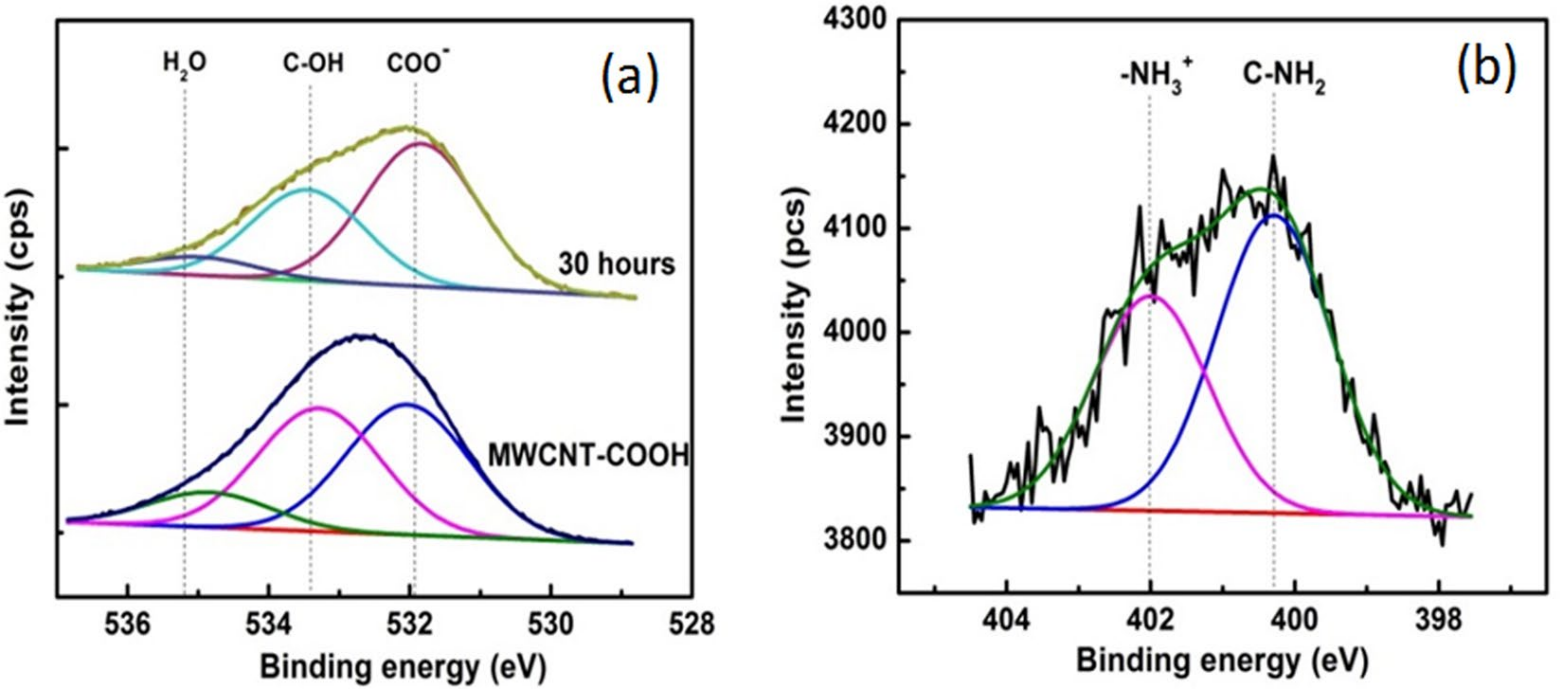
The XPS deconvolution spectra of the: (**a**) O1s and (**b**) N1s core level region of MWCNTs functionalized with thiol using cysteaminium.

In the thiolation of oxi-MWCNTs by -COO^−^/NH_3_^+^ *zwitterionic* acid-base reaction in water medium, the negative charges of COO^−^ groups may bind with NH_2_ of the cysteamminium chloride by the electrostatic attraction^39,40^. A quantitative analysis of the reaction by following the changes of COO^−^ groups and ionic interaction with NH_3_^+^, was performed by XPS measurements (Fig. 6).

The binding energy was calibrated by referencing the C1s peak at 284.6 eV using XPS data. Detailed information on surface oxygen functional groups was obtained after analyzing the O1s spectrum (Fig. 6a). The peak centered at ~532 eV was attributed the oxygen double bond in the carboxylic acid, (O*=C)–O^−^. The hydroxyl group of carboxylic group was denoted as C–OH, and was centered at about 533.4 eV. Interestingly, the quantitative calculation via XPS results shows a difference in terms of peak ratio of these two bands during the thiolation. It raised from about 1.02 for oxi-MWCNTs to 1.63 for MWCNTs-COO^−^/^+^NH_3_(CH_2_)_2_SH after 30 h of reaction. The reduction of C-OH in the thiolated MWCNTs can be explained as follows: negatively charged CNTs were attracted to the positively charged amine groups of cysteaminium during the reaction process. The N1s core lines of MWCNTs functionalized with cysteaminium after 30 h of reaction are shown in Fig. 6b. The N1s core lines exhibit two new peaks at around 400.3 and 402 eV, assigned to -C-NH_2_^41,42^ and -NH_3_^+^^17–19,42^, respectively (see complementary information, Fig. S3)^43^.

**Figure 7:**
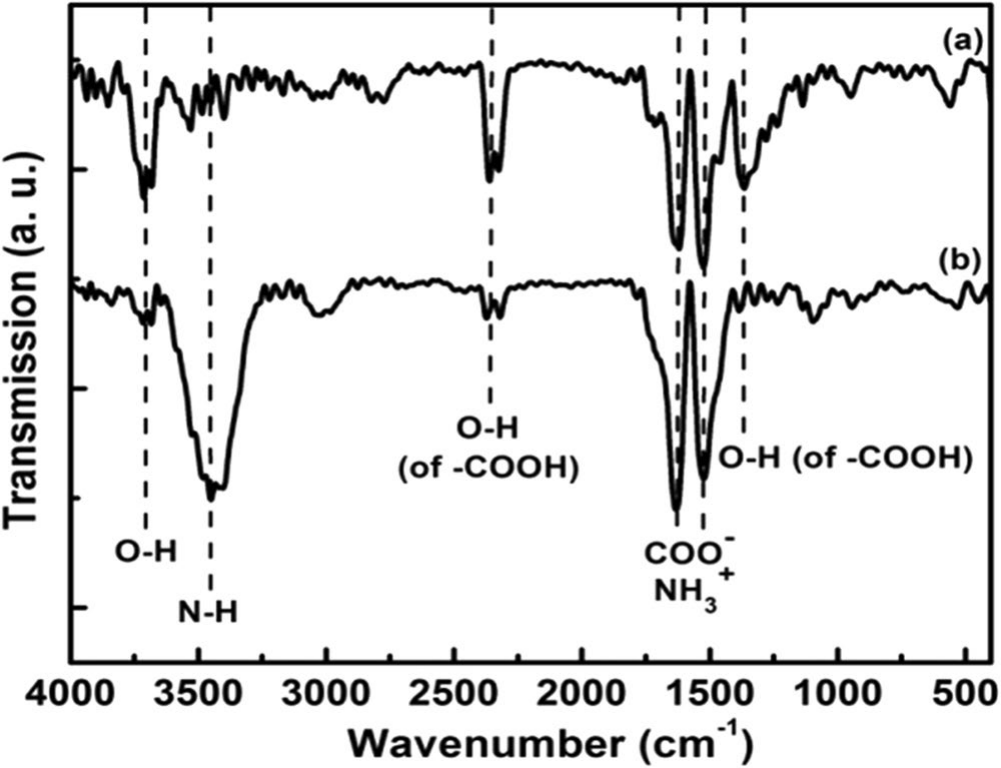
FT-IR spectra of: (**a**) the acid treated and (**b**) thiolated MWCNTs.

Figure 7 displays FTIR spectra of oxi-MWCNTs and thiolated MWCNTs samples. As it can be seen from this figure that, main peaks assigned to hydroxyl and carboxyl groups are detectable in the FTIR spectrum of the oxi-MWCNTs. For the thiolated-MWCNTs, a new peak located at the 3450 cm^−1^ band was observed. This can be due to the N-H symmetrical amine vibration. Two broader bands at around 1630 cm^−1^ and 1524 cm^−1^, assigned to the overlapping of the COO^−^ bands and NH_3_^+^ groups, are also increased for thiolated MWCNTs. These two bands suggest that thioalted MWCNTs contain protonated amino groups, forming *zwitterionic* species via the deprotonation of carboxyl groups (-COO^−^). These results confirm the successful grafting of thiol groups on the MWCNTs surface.

### Synthesis of AuNPs decorated CNTs

**Figure 8:**
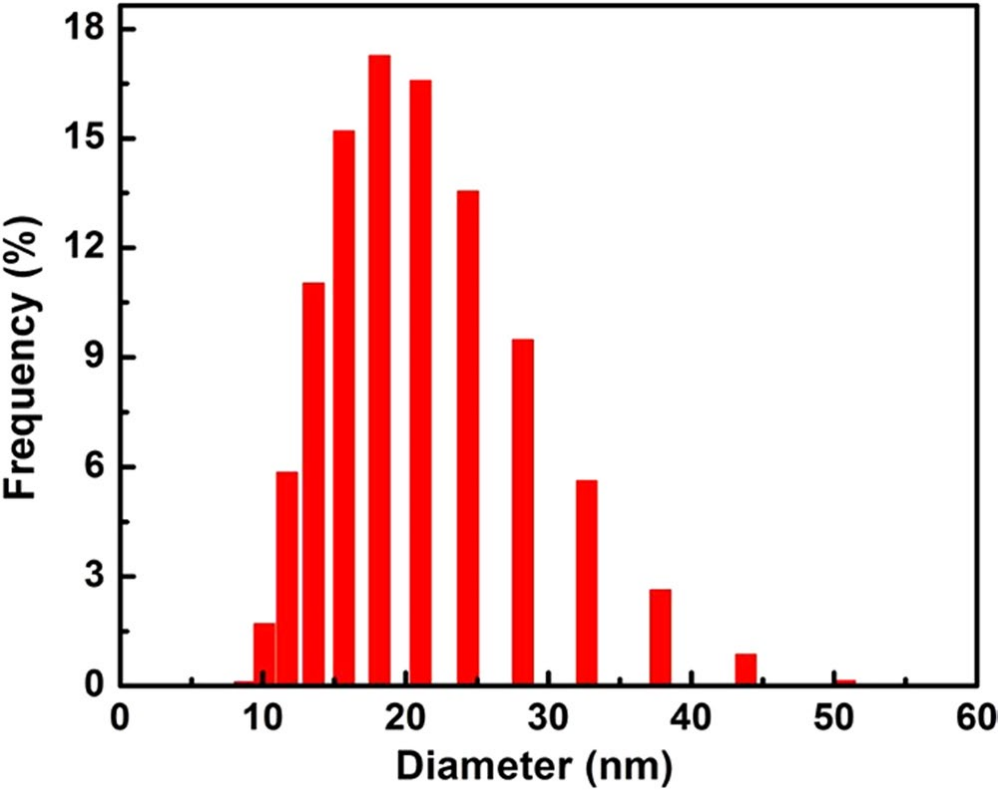
Size distributions of gold nanoparticles on the MWCNTs.

To prepare AuNPs decorated MWCNTs hybrid nanoparticles, gold nanoparticles were synthetized according to the experiments described in section 2.2 in which AuNPs were obtained from HAuCl_4_ solution by a reduction method. Figure 8 shows the particle size distribution histogram obtained from the SEM image and dynamic light scattering analysis, it can be seen that the gold NPs are spheroidal and particle size distribution ranges mainly in the order of 15–35 nm.

**Figure 9:**
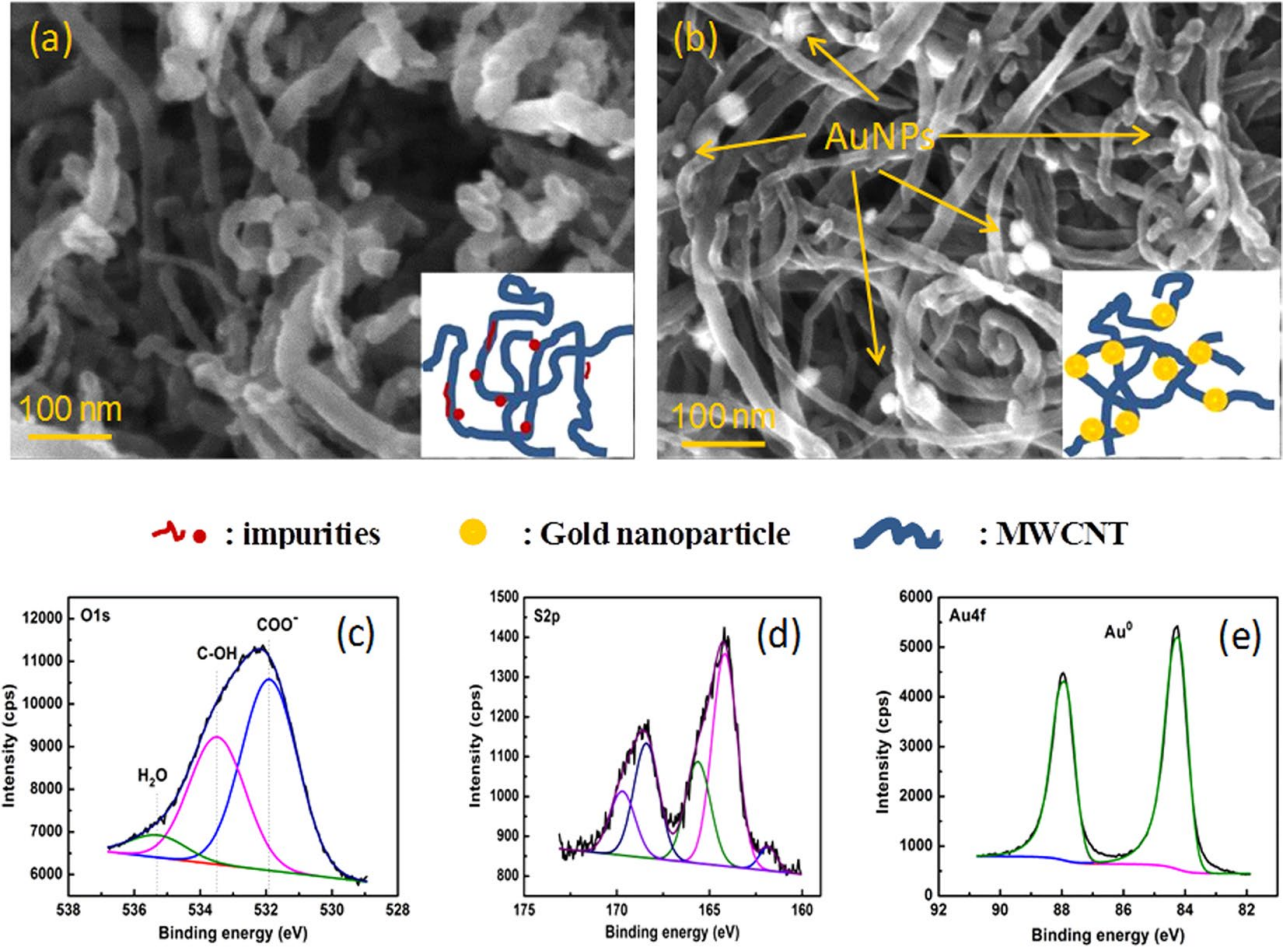
SEM images of (**a**) thiolated- MWCNTs and (**b**) AuNPs-MWCNT. showing the presence of AuNPs decorated on the MWCNTs surface (arrows) and core lines devolution of XPS spectra for: (**c**) O1s; (**d**) S2p and (**e**) Au4f. The inserted images illustrate the structure of AuNPs-CNTs.

The grafting functionalized CNTs using gold NPs solution is then performed. Figures 9a and 8b show SEM images of thiolated MWCNT (a) and AuNPs decorated MWCNTs, respectively. It is clearly seen that several isolated gold NPs on the MWCNTs surface can be observed. They are well distributed on the whole MWCNTs. These morphological observations were supported by XPS results, as shown in Fig. 9c–e. The O1s core line still shows three components assigned to carboxylic group (component 1), C-OH (component 2), H_2_O residue (component 3). The S2p core line was split into two spin-orbit doublets, assigned to different chemical bonds: the first doublet 1 (164.2 and 165.6 eV) refers to free thiol (S-H), while the second doublet (168.4 and 169.6 eV) can be assigned to H_2_SO_4_ residuals from MWCNT preprocessing. Finally, the component at 161.8 eV was associated to the presence of S-Au bond. Conversely, the Au 4 f peak falls at 84.22 eV, slightly shifted with respect to the Au^0^ position due to the presence of Au-S covalent bond in agreement with previous studies.

The atomic concentrations of the treated MWCNTs have been quantitatively calculated from XPS spectra and results were summarized in Table 1.

**Table 1:** Composition of the functionalized and Au decorated MWCNTs.

	C1s	O1s	N1s	S2p	Au 4 f
Oxidized MWCNTs	90.16	8.69	//	1.1	//
Thiolated MWCNTs	93.7	4.9	1.3	0.9	//
Gold NPs decorated with MWCNTs	90.88	6.5	1.2	1.0	0.4

These results show clearly that the modifications lead to the reduction of atomic oxygen and the N1s were clearly observed in the thiolated MWCNTs while the XPS peak positions demonstrate that the decoration of gold NPs occurs spontaneously with the formation of Au-S bonds. The latter confirmed the successful synthesis of the AuNPs decorated MWCNTs by chemical bonds and thus expected to have robust surface for specific applications such as biosensors or biomolecules sensors.

**Figure 10:**
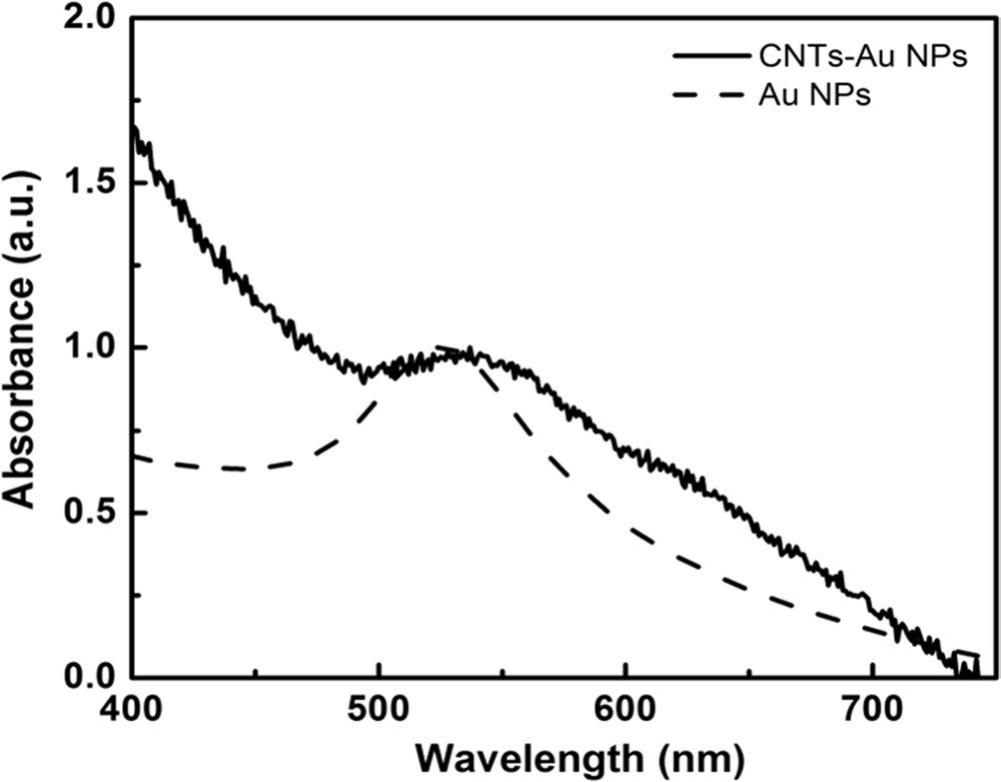
UV-Visible absorption spectra of gold NPs decorated with MWCNTS and pure gold NPs solution.

Figure 10 shows the absorption spectrum of pure gold NPs solution and the as-prepared gold NPs-MWCNT nanocomposites. The typical absorption band at 525 nm, found in the UV-Vis spectrum of pure gold NPs, was due to its Surface Plasmon Resonance (SPR). In the case of Au NPs decorated with MWCNTs, a sensible broadening and red-shift of the Plasmon resonance band was observed which can be attributed to covalent bonds between gold NPs and the walls of MWCNTs. The spectral shoulder at around 625 nm may be associated to gold NPs aggregates bonded to the MWCNT walls.

## Conclusions

In summary, this paper shows our contribution on the synthesis of new thiol-functionalized MWCNTs by using covalent ionic bonds approach in which the strong electrostatic attractions between negatively charged oxi-MWCNTs and positively charged cysteamine were reacted to form stable covalent chemical bonds. Further decoration of functionalized MWCNTs with gold nanoparticles was achieved by a simple reaction of thiol groups in the cysteamine with Au cation. In all cases, the reactions have been proven by both spectroscopic (Raman, UV-Vis, FT-IR) and morphological tools (SEM, TEM). The deconvolution of XPS shows to be useful to confirm the chemical states of atomic elements for each synthetic step and is a complementary tool to support the obtained results from other analytical methods. Our functionalization approach demonstrates the possibility to obtain a good AuNPs chemically linked MWCNTs by a simple and greener method without using any dangerous chemicals as previously reported in the literature.

## Supplementary information

Supplementary information
