# Supplementary material for: Synthesis of Gold Nanoparticles Decorated with Multiwalled Carbon Nanotubes (Au-MWCNTs) via Cysteaminium Chloride Functionalization
Source: Sci Rep. 2019 Apr 5;9:5667. doi: 10.1038/s41598-019-42055-7 (PMC6450879; doi:10.1038/s41598-019-42055-7)
Supplement: Supplementary file 1 — Supplementary information [file 41598_2019_42055_MOESM1_ESM.docx]

Supplementary information

Synthesis of Gold Nanoparticles Decorated with Multiwalled Carbon Nanotubes (Au-MWCNTs) via Cysteaminium Chloride Functionalization

Vu Duc Chinh*^a,b,c^, Giorgio Speranza^d^, Claudio Migliaresi^a^, Nguyen Van Chuc^b^, Vu Minh Tan^e^ and Nguyen-Tri Phuong^f,g*^

^a^ Department of Industrial Engineering, University of Trento, via Sommarive 9, 38123 Trento, Italy.

^b^ Institute of Materials Science, Vietnam Academy of Science and Technology, 18 Hoang Quoc Viet road, Cau Giay district, Hanoi, Vietnam.

^c^ Graduate University of Science and Technology, Vietnam Academy of Science and Technology, 18 Hoang Quoc Viet road, Cau Giay district, Hanoi, Vietnam.

^d^ Centre for Materials and Microsystems, Fondazione Bruno Kessler, via Sommarive 18, I-38123, Povo (Trento), Italy.

^e^ Hanoi University of Industry, Bac Tu Liem, Hanoi, Vietnam.

^f^ Institute of Research and Development, Duy Tan University, Da Nang 550 000, Viet Nam

^g^ Department of Chemistry, Université de Montréal, Quebec, Canada.

Corresponding author: [Phuong.nguyen.tri@umontreal.ca](mailto:Phuong.nguyen.tri@umontreal.ca); Tel: + 514-340 5121 (7326)

FT-IR analysis was conducted on pristine and functionalized MWCNTs after various treatments, in the range of 400 to 4000 cm^-1^. Figure 5 shows IR spectra of the pristine MWCNTs with a broad peak at 3713 cm^‑1^, assigned to the O-H stretching in hydroxyl groups (Figure 5a) of the carboxyl groups. The initial low content of carboxyl groups on the surface of pristine MWCNTs could be attributed to the partial oxidation of the surfaces of MWCNTs during preparation and purification steps. The acidic treatment of MWCNTs leads to an increasing of the peak intensity at the 1624 cm^-1^ band, assigned to the C=O stretching indicating that carboxylic groups are formed on the surface of the MWCNTs (Figure 5b). The implantation of functional groups leads to the increasing also the peak at 2361 cm^−1^ associated to strongly hydrogen-bonded in the COOH group ^41^ and -OH bending modes at 1364 cm^-1^ band. It is worth noting that this peak can be affected by water vapor or carbon dioxide. However, this effect has been eliminated by reducing the time between the background measurements in combination with the purging sample chamber by high-purity nitrogen. These results are in good agreement with those obtained by XPS results as previously described.


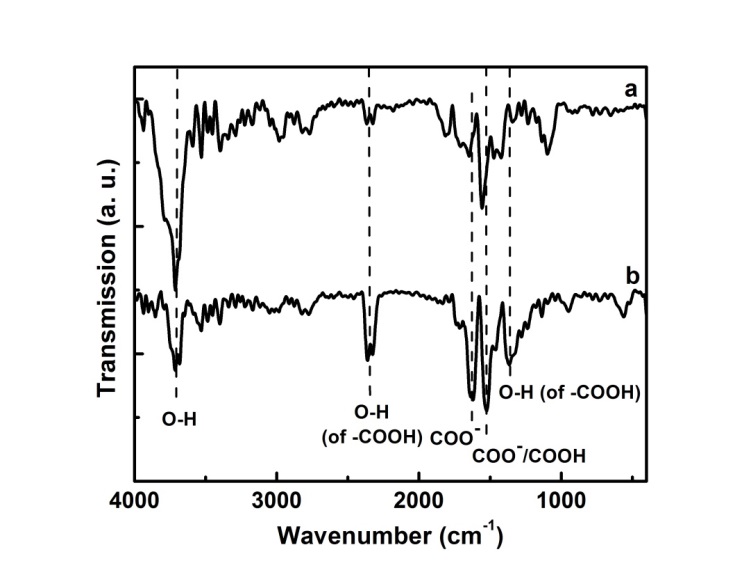


**Figure S1**  FT-IR spectra of: a) the pristine MWCNTs and b) the acidic treated MWCNTs


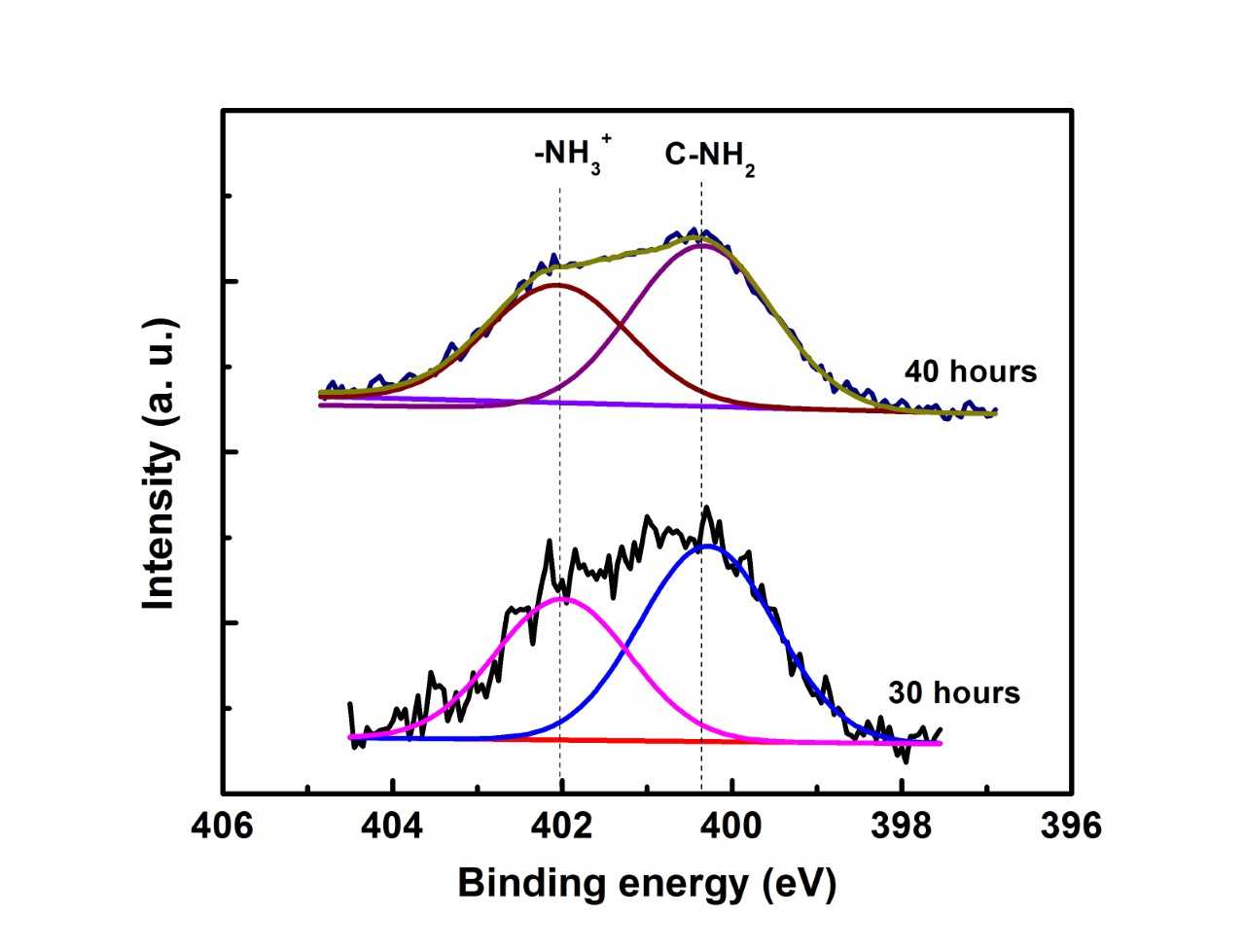


**Figure S2**  XPS spectra of the MWCNTs thiolated at different times.


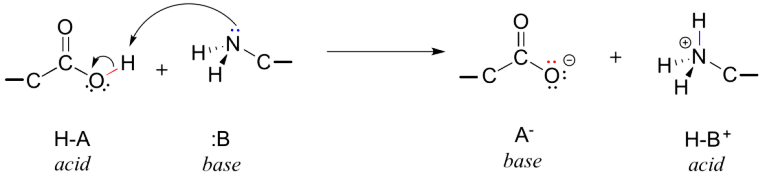


**Figure S3:** Possible mechanis of the thiolation reactionbetween the negatively charged CNTs with positively charged amine groups of cysteaminium
